# Validity and reliability of the Arabic version of the Self-Efficacy for Managing Chronic Disease scale in rheumatoid arthritis patients

**DOI:** 10.1007/s10067-022-06183-2

**Published:** 2022-06-15

**Authors:** Shymaa A. Sarhan, Doaa E. Kamal, Mona S. Hamed, Dalia I. Mostafa

**Affiliations:** 1 Department of Rheumatology and Rehabilitation, Faculty of Medicine, Zagazig University, Zagazig, Egypt; 2 Community Department at Faculty of Medicine, Zagazig University, Zagazig, Egypt

**Keywords:** Rheumatoid arthritis, SEMCD questionnaire, Validity

## Abstract

**Background and objectives:**

Rheumatoid arthritis (RA) is an autoimmune inflammatory condition that causing disability and affection of patient’s quality of life (QoL). Self-efficacy investigation helps us to detect the requirements of chronically affected patients and evaluation of self-care management programs. The aim of our study was to test validity and reliability of Self-Efficacy for Managing Chronic Disease (SEMCD-Arabic) in RA patients.

**Method:**

This study included 248 RA patients, carried out at Rheumatology and Rehabilitation Department. The SEMCD-Arabic Validity was assessed by correlating the SEMCD-Arabic scale with the validated Arabic version of the modified Health Assessment Questionnaire HAQ (MHAQ), the Arabic version of the Multidimensional Assessment of Fatigue (MAF) scale, and the Arabic version of Short Form 36 version 2 for quality of life (SF QoL). Internal consistency, test–retest reliability was assessed.

**Results:**

Convergent validity was confirmed by a positive correlation between (physical, mental) component of SF QoL and SEMCD-Arabic (*r* = 0.918, *r* = 0.925) respectively, and negative correlation between MAF and SEMCD-Arabic (*r* =  − 0.657) and MHAQ with SEMCD-Arabic (*r* =  − 0**.**595). Discriminant validity confirmed by a significant negative correlation between visual analogue scale (VAS) for pain, disease activity scale (DAS28), Morning stiffness, patient health, physician health, age, duration, and SEMCD-Arabic (*r* =  − 0.1–0.7) (*P* < 0.001). Test–retest reliability was estimated which revealed a high interclass correlation coefficient (ICC = 0.87–0.997) indicating excellent agreement and internal consistency is acceptable as the Cronbach’s alpha value (0.660 to 0.78).

**Conclusion:**

The SEMCD-Arabic questionnaire can be used as a valid and reliable measure for assessment of patient’s self-efficacy in RA.

**Table:** 

**Key Points** *• The SEMCD-Arabic questionnaire has a statistically significant validity in correlation with different clinical manifestations MHAQ, SF QoL, and MAF.* *• The Arabic SEMCD is highly reliable with a Cronbach’s alpha of 0.660 to 0.78.*	

## Introduction

Rheumatoid arthritis (RA) is a known autoimmune chronic inflammatory condition that affecting the joints by pain and swelling and consequently results in marked disability and affection of patient’s quality of life (QoL). RA patients need long course of treatment with a great effects on patients and their families economically and psychologically [1]. RA frequently affects walking dressing, meal preparation, personal care, and eating as activities of daily living [2].

At least three quarters of RA patients have fatigue even though presence of approved treatments for RA. Fatigue can be due to many causes; chronic pain, poor mood, sleeps disturbance, and other comorbidities are associated with patient’s fatigue. RA patients feel tired or even exhausted due to high inflammatory markers, cytokines that are frequently elevated; therefore, disease activity is often causing fatigue [3].

Female gender, pain and anxiety, current medications, and obesity are factors associated with severe fatigue in RA patients rather than physical capacity [4, 5].

Self-efficacy is a psychological concept that widely used nowadays and it means patient’s belief/perception in his/her capacity to perform their activities of daily living. Self-efficacy is considered now as an important issue in self-care management programs for patients with a chronic illness [6]. These programs can be presented as online group forms which include different skills for problem solving and provide good contact with healthcare members [7].

Self-efficacy investigation in patients with chronic and debilitating illness such as RA helps us to detect the requirements of the chronically affected patients and consequently planning and evaluation of self-care management programs suitable for those patients [8].

Self-Efficacy for Managing Chronic Disease (SEMCD) scale has been psychometrically used widely in chronic illnesses. SEMCD-Eng has been used extensively to evaluate self-efficacy of many chronic diseases as systemic sclerosis (SSc), arthritis, diabetes, heart and lung diseases, and their self-management programs [9]. As far as we know, SEMCD scale in English version only validated in arthritis not in RA disease by name; therefore, the aim of our study was to detect whether the SEMCD-Arabic scale can be used as a valid and reliable measure for self-efficacy in RA patients?

## Subjects and methods

### Patients

In the current cross-sectional study, 248 RA patients were included according to the American College of Rheumatology/European League Against Rheumatism new RA criteria [10], and it was carried out at Rheumatology and Rehabilitation Department. The study sample calculated using open epi -I program assuming that the total attendee was 700 RA patients at 50% self-efficacy in controlling their disease and 5% margin of errors, their age was ≥ 18 years, with one year or more disease duration, and they could understand and speak Arabic. Patients with significant cognitive impairment or severe psychiatric illness which interfere with the assessment were excluded from the study. All study participants gave their informed consent, and the research protocol number (ZU-IRB#9026–13-10–2021) was authorized by the local Institutional Review Board (Zagazig University, Egypt), in accordance with The Code of Ethics of the World Medical Association (Declaration of Helsinki 1964) for studies involving humans.

### Clinical assessment

Data were collected from clinical history, general examination, and musculoskeletal examination, which included patient’s age, disease duration, pain by VAS (0–100), morning stiffness, and patient and physician global health assessment by VAS (0–10). Disease activity is determined by disease activity scale (DAS28) [11].

### Patient-reported outcome measures (PROMs)

#### The Self-Efficacy for Managing Chronic Disease scale

The 6-item SEMCD assesses participants’ self-efficacy (confidence) in preventing physical discomfort, fatigue, pain, mental distress, and other symptoms interfering with their goals. It also analyses confidence in order to reduce the need to visit a doctor and the effect of the disease on daily activities. The overall score is obtained by summing the items’ scores, which range from 1 (not at all confident) to 10 (very confident) [12].

The SEMCD was translated from English to Arabic by a professional translator who is fluent in the Arabic language. The original developer agreed to use the translation [13].

#### The modified Health Assessment Questionnaire HAQ (MHAQ)

It is a self-applied outcome questionnaire, developed as a simplified version of the HAQ, that is used in RA patients to evaluate patient satisfaction with daily activities as well as perceived changes in level of difficulty [14].

Dressing, grooming, arising, eating, walking, hygiene, reaching, gripping, and chores are among the eight items in the eight categories. These activities are scored on a 4-point scale, with 0 indicating little difficulty, 1 indicating moderate difficulty, 2 indicating great difficulty, and 3 indicating inability to complete. Higher scores imply worse function and greater disability [15].

#### The Multidimensional Assessment of Fatigue (MAF) scale

Belza et al. created the MAF scale for older persons with RA. This 16-item scale is a self-administered questionnaire that measures 4 aspects of fatigue: degree and intensity, amount of distress resulted from it, timing, and the degree to which fatigue interferes with everyday activities throughout the previous week [16]. To calculate the Global Fatigue Index (GFI), first multiply the rating score of item 15 (1–4) by 2.5 to convert it to a 10-point scale. The GFI is then determined using the following formula: GFI score = the sum of the rating scores for items 1–3 + items 4–14 + have an average rating score of 4–14 + . Item 15 now has a new score. The GFI does not include item 16 [17]. The GFI scale runs from 1 to 50 (1 = no fatigue, 50 = extreme fatigue) [18].

In patients with RA [6] and ankylosing spondylitis [8], the MAF has demonstrated high reliability and validity. It was used to assess fatigue in RA patients in its Arabic version [9].

#### Short Form 36 QoL version 2 (SF QoL)

It is a 36-component self-administered health assessment that rates QoL on eight scales: overall health, physical functioning, physical role, pain, vitality, social functioning, emotional difficulties, and mental health. The score on each scale ranges from 0 to 100. Higher ratings indicate a higher quality of life. The eight scales are summarized into physical and mental component score [19].

### Construct validity

*Convergent validation* was assessed by correlating the Arabic SEMCD version with the scores of the Arabic version of the Multidimensional Assessment of Fatigue scale, the modified Health Assessment Questionnaire, and the Short Form 36 QoL Questionnaire.

*Discriminant validity* was evaluated by correlating the SEMCD scale with the disease activity (DAS-28), patient’s age, disease duration, pain by VAS (0–100), morning stiffness, and patient and physician global health assessment by VAS (0–10).

### Internal consistency, test–retest reliability

Two interviews with the same interviewer, 2 weeks apart, were used to examine test–retest reliability. The interclass correlation coefficient (ICC) is used to assess agreement between repeated interviews, with an ICC of 0.7 or higher indicating strong agreement [9]. Internal consistency was measured by Cronbach’s alpha coefficient.

### Statistical analysis

The collected data was analyzed using the Statistical Package for Social Science (SPSS) program version 26. Qualitative data were represented as number and frequencies and quantitative variables were represented as mean ± standard deviation (SD) and median (IQR) (for not normally distributed data). Reliability and the absolute agreement between test–retest were evaluated by calculating “intraclass correlation coefficient *r*” regarding the questions and the total SEMCD score. Spearman’s correlation (*r*) was used to correlate between the different variables. Positive correlation coefficient (*r*) values indicate positive association between the variables, and negative (*r*) values indicate negative associations; correlation is considered strong if *r* > 0.7, and weak correlation if *r* ≤ 0.29. Statistical significance and highly statistical significance when the significant probability (*P* value) were < *0.05* and < *0.001* respectively.

## Results

The mean age of the studied group was 44.5 ± 12.2 ranging from 22 to 70 years, majority of cases were female (90.3%), only 8.1% were smoker, and most of cases (93.5%) were married. More than half of cases (72.5%) did not work. Most of them live in village (62.9%) and only 37.1% live in city.

Regarding comorbidities, 16.1% of cases were hypertensive, 8% were diabetic, and 3.2% had ischemic heart disease and IL. Majority of cases were on regular medication (85.5%). The median (IQR) duration of disease was 6 (3–11) years, the median (IQR) VAS for pain is 60 (40–70), and the median (IQR) of DAS is 4.6 (3.5–6.1); regarding MHAQ, more than half (53.2%) were mild grade (Table 1).

**Table 1:** Basic characteristics and patient-reported outcome measures of the studied group (*n* = 248)

Variable		*N*	%
**Age (years)**	Mean ± SD Range	44.5 ± 12.2 (22–70)	
**Sex**	•Male •Female	24 224	9.7 90.3
**Smoking habit:**	•Smoker	20	8.1
**Marital status:**	•Married •Single	232 16	93.5 6.5
**Occupation:**	•Working	68	27.4
**Residence**	•City •Village	92 156	37.1 62.9
**Comorbidities**	•HTN	40	16.1
	•Diabetes	20	8
	•HCV	4	1.6
	•HV	4	1.6
	•Ischemic heart	8	3.2
	•Il	8	3.2
**Regular medications**	•No	36	14.5
	•Yes	212	85.5
**Drugs**	**HDQ**	200	80.6
	**MTX**	136	54.8
	**LEF**	116	46.8
	**SSZ**	60	24.2
	**AZA**	12	7.1
	**Steroids**	136	54.8
	**NSAIDS**	20	11.6
	**Analgesics**	40	23.3
	**Vitamins**	112	65.1
	**Others**	17	6.9
**Disease duration**	Median (IQR)	6 (3–11)	
**VAS for pain**	Median (IQR)	60 (40–70)	
**DAS**	Median (IQR)	4.6 (3.5–6.1)	
**Patient global health**	Median (IQR)	5 (3–7)	
**Physician global health**	Median (IQR)	4.6 (3.5–6.1)	
**Morning stiffness (min)**	Median (IQR)	15 (5–30)	
**MHAQ**	Median (IQR)	1 (0.64–1.37)	
**MHAQ grading**	Normal Mild Moderate Severe	40 (16.1) 132 (53.2) 48 (19.4) 28 (11.3)	

Regarding MAF score domains, the median (IQR) of degree is 7 (5–8), severity is 7 (5–8), distress is 7 (5–8), activity is 6.1 (4.4–6.8), timing is 0.75 (0.5–1), and MAF (total score) is 27.9 (20.9–32.2). Regarding quality-of-life domain and total score, median (IQR) of physical function was 45 (30–60); median (IQR) of each of limitation of physical health, emotional problem, fatigue, and emotional wellbeing is 40 (30–60); median (IQR) of social, pain, general health, and total physical is 40 (30–50); and median (IQR) of total mental is 42.5 (30–57.5) (Table 2).

**Table 2:** MAF and QoL among the studied patients

Items		Mean ± SD Median (IQR)
**MAF**	Degree	6.73 ± 2.23 7 (5–8)
	Severity	6.53 ± 2.16 7 (5–8)
	Distress	6.53 ± 2.29 7 (5–8)
	Activity	5.83 ± 2.06 6.1 (4.4–6.8)
	Timing	0.69 ± 0.27 0.75 (0.5–1)
	MAF (total score)	26.35 ± 8.37 27.9 (20.9–32.2)
**QoL**	Physical function	47.26 ± 17.9 45 (30–60)
	Limitation of physical health	42.26 ± 18.03 40 (30–60)
	Emotional problem	43.55 ± 19.8 40 (30–60)
	Fatigue	44.68 ± 19.1 40 (30–60)
	Emotional wellbeing	43.71 ± 18.2 40 (30–60)
	Social	41.77 ± 16.64 40 (30–50)
	Pain	39.84 ± 15.32 40 (30–50)
	General health	42.09 ± 15.6 40 (30–50)
	Total physical	42.86 ± 14.88 40 (30–50)
	Total mental	43.43 ± 16.7 42.5 (30–57.5)

### Reproducibility

Patients were tested for differences in test–retest scores and internal consistency. The repeatability of each item on the QoL questionnaire was strong, with little variation between domains where the interclass correlation coefficient was high (ICC) values ranging from 0.87 to 0.997 indicating excellent agreement. Cronbach’s alpha for the SEMCD was 0.946 representing an acceptable internal consistency. The item-total correlations ranged from 0.811 to 0.917 indicative to a good reliability range (Table 3).

**Table 3:** Self-Efficacy for Managing Chronic Disease 6-Item Scale (SEM6S) and interclass correlation coefficient of the test–retest values

Characteristic	Mean ± SD Median (IQR)	Item-total correlation	ICC (CI 95%)	Alpha
Q1: How confident do you feel that you can keep the fatigue caused by your disease from interfering with the things you want to do?	4.94 ± 2.15 5 (4–6)	0.896	0.997 (0.996–0.997)	0.998
Q2: How confident do you feel that you can keep the physical discomfort or pain of your disease from interfering with the things you want to do?	4.69 ± 2.06 5 (3–6)	0.899	0.996 (0.995–0.997)	0.998
Q3: How confident do you feel that you can keep the emotional distress caused by your disease from interfering with the things you want to do?	4.47 ± 2.2 4 (3–6)	0.840	0.992 (0.99–0.994)	0.996
Q4: How confident do you feel that you can keep any other symptoms or health problems you have from interfering with the things you want to do?	4.35 ± 1.96 4 (3–5)	0.917	0.871 (0.838–0.898)	0.931
Q5: How confident do you feel that you can the different tasks and activities needed to manage your health condition so as to reduce your need to see a doctor?	4.5 ± 2.2 4 (3–5)	0.811	0.982 (0.976–0.986)	0.991
Q6: How confident do you feel that you can do things other than just taking medication to reduce how much your illness affects your everyday life?	5.15 ± 2.89 4 (3–8)	0.816	0.995 (0.994–0.996)	0.998
Total	4.68 ± 1.93 4.7 (3.3–6.2)	––––-	0.994 (0.992–0.995)	0.997

Cronbach *α* = 0.946, interclass correlation coefficient (confidence interval)

### Discriminant validity

There was statistically significant strong negative correlation between VAS for pain, DAS, morning stiffness, patient global health, physician global health, and all SEMCD questions. Also there was statistically significant negative correlation between age and Q1, Q3, Q4, Q5, and total score of SEMCD and a significant negative correlation between duration and confidence to do things other than just taking medication to reduce illness affects everyday life (Table 4).

**Table 4:** Correlation of SEM6S and different parameters

SEM6S	Age	Duration	VAS for pain	DAS	MHAQ score	Morning stiffness	Patient health	Physician health
Q1	− 0.180**	− 0.053	− 0.625**	− 0.559**	− 0.488**	− 0.475**	− 0.667**	− 0.624**
Q2	− 0.085	− 0.092	− 0.569**	− 0.518**	− 0.401**	− 0.425**	− 0.614**	− 0.588**
Q3	− 0.126*	0.043	− 0.630**	− 0.642**	− 0.474**	− 0.497**	− 0.657**	− 0.641**
Q4	− 0.223**	0.000	− 0.612**	− 0.639**	− 0.559**	− 0.509**	− 0.682**	− 0.685**
Q5	− 0.129*	− 0.120	− 0.483**	− 0.546**	− 0.524**	− 0.250**	− 0.481**	− 0.472**
Q6	− 0.130	− 0.183**	− 0.604**	− 0.643**	− 0.548**	− 0.554**	− 0.603**	− 0.665**
Total	− 0.174**	− 0.111	− 0.693**	− 0.695**	− 0.595**	− 0.520**	− 0.704**	− 0.731**

^*^Significant *P* < .05

^**^Highly significant *P* < .001

### Convergent validity

**Fig. 1:**
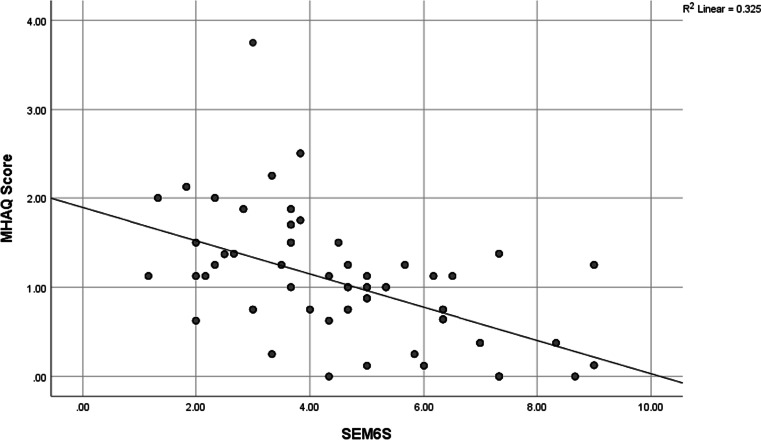
Correlation of SEM6S and MHAQ scores

**Fig. 2:**
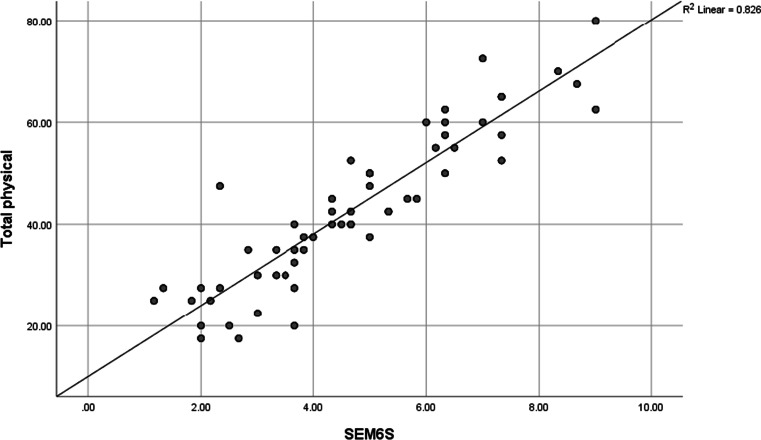
Correlation of SEM6S and total physical item of quality of life

**Fig. 3:**
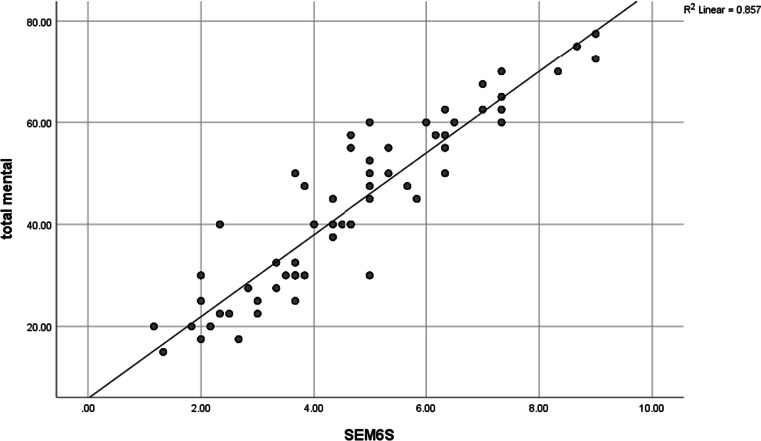
Correlation of SEM6S and total mental item of quality of life

A statistically significant negative correlation between MHAQ and SEMCD was found (Fig. 1) where there was moderate negative correlation between MHAQ score and Q4, Q5, Q6, and total score of SEMCD-Arabic (*r* =  − 0.559**, *r* =  − 0.524**, *r* =  − 0.548**, *r* =  − 0.595**) respectively. There was statistically significant strong positive correlation between SEM6S and all items of quality-of-life score (Table 5, Figs. 2 and 3) with a strong positive correlation between total (physical, mental) component of SF 36 and SEMCD-Arabic (*r* = 0.918**, *r* = 0.925**) respectively. There was statistically significant negative correlation between degree, severity, distress, timing, MAF total, and all SEMCD questions. Also, our results revealed a strong significant negative correlation between degree, severity, distress, MAF total, and all SEMCD-Arabic questions (*r* =  − 0.708**, *r* =  − 0.677**, *r* =  − 0.673**, *r* =  − 0.657**), with activity negatively correlated with Q1, Q2, Q3, Q4, and total score of SEMCD-Arabic (*r* =  − 0.529**) (Table 6).

**Table 5:** Correlation of SEM6S and quality of life scores

Characteristic	QOL physical function	Limitation of physical health	Emotional problem	Fatigue	Emotional wellbeing	Social	Pain	General health
Q1	0.748**	0.800**	0.829**	0.858**	0.627**	0.790**	0.742**	0.589**
Q2	0.685**	0.799**	0.805**	0.847**	0.570**	0.744**	0.714**	0.615**
Q3	0.666**	0.721**	0.744**	0.762**	0.477**	0.699**	0.655**	0.551**
Q4	0.784**	0.829**	0.829**	0.849**	0.626**	0.759**	0.764**	0.612**
Q5	0.736**	0.69**	0.73**	0.76**	0.53**	0.7**	0.63**	0.498**
Q6	0.752**	0.779**	0.778**	0.828**	0.657**	0.678**	0.638**	0.489**
Total	0.860**	0.911**	0.926**	0.962**	0.689**	0.838**	0.799**	0.652**

^*^Significant *P* < .05

^**^Highly significant *P* < .001

**Table 6:** Correlation of SEM6S and MAF

Items	Degree	Severity	Distress	Activity	Timing	MAF total
Q1	− 0.607**	− 0.586**	− 0.583**	− 0.517**	− 0.347**	− 0.622**
Q2	− 0.662**	− 0.628**	− 0.554**	− 0.506	− 0.490**	− 0.636**
Q3	− 0.629**	− 0.622**	− 0.655**	− 0.608**	− 0.390**	− 0.682**
Q4	− 0.605**	− 0.611**	− 0.601**	− 0.540**	− 0.454**	− 0.643**
Q5	− 0.556**	− 0.528**	− 0.485**	− 0.444**	− 0.301*	− 0.560**
Q6	− 0.589**	− 0.543**	− 0.579**	− 0.294*	− 0.253*	− 0.554**
Total	− 0.708**	− 0.677**	− 0.673**	− 0.529**	− 0.418**	− 0.657**

^*^Significant *P* < .05

^**^Highly significant *P* < .001

## Discussion

RA as a chronic inflammatory disease markedly affects the QoL of the patient as it frequently causes joint damage, pain, fatigue, functional impairment [18], and disability that extensively affecting activity of RA patients; therefore, it is of great interest to assess self-efficacy for managing chronic diseases to detect patients’ requirements for help and to assess self-management programs [20]. Therefore, in our study, we evaluated the SEMCD-Arabic as a valid and reliable scale in RA patients aiming at proper self-efficacy management of that chronic disease in an Arabic population.

The results of our study revealed that the SEMCD-Arabic can be used as a valid and reliable scale in assessment of self-efficacy in management of RA. The results support the content validity, construct validity, internal consistency, and test–retest reliability of the SEMCD-Arabic for patients with RA.

As regards the construct validity of the SEMCD-Arabic, our results revealed a strong convergent validity of SEMCD-Arabic compared with the SF 36v2, MAF, and MHAQ scores, as we found a strong positive correlation between total (physical, mental) component of SF 36v2 and SEMCD-Arabic (*r* = 0.918**, *r* = 0.925**) respectively. Also, we found that degree, severity, distress, and MAF total correlated negatively with all SEMCD-Arabic questions (*r* =  − 0.708**, *r* =  − 0.677**, *r* =  − 0.673**, *r* =  − 0.657**), with activity also negatively correlated with Q1, Q2, Q3, Q4, and total score of SEMCD-Arabic (*r* =  − 0.529**). Moreover, our results found moderate negative correlation between MHAQ score and Q4, Q5, Q6, and total score of SEMCD-Arabic (*r* =  − 0.559**, *r* =  − 0.524**, *r* =  − 0.548**, *r* =  − 0.595**).

Our results were similar to Mattsson et al. [9] who found a moderate correlations between the SEMCD-Swe and physical and mental aspects of HRQoL in SSc patients (RAND-36 *r* = 0.53, *P* < 0.001), fatigue (MAF, *r* =  − 0.59, *P* < 0.001), and disability (HAQ-DI, *r* =  − 0.55, *P* < 0.001).

Also Alkabeya et al. reported similar results as regards validation of the Arthritis Self-Efficacy Scale-8 (ASES-8) scores with RA disease-related variables in Arabic population, which is scale for a patient-reported arthritis-specific self-efficacy [21].

As regards discriminant validity of the SEMCD-Arabic, a significant negative correlation was found between VAS for pain, DAS, morning stiffness, patient health, physician health, and all SEMCD questions (*r* =  − 0.693**, *r* =  − 0.695**, *r* =  − 0.520**, *r* =  − 0.704**, *r* =  − 0.731**); also, we found a negative significant correlation between age and Q1, Q2, Q3, Q4, Q5, and total score of SEMCD-Arabic (*r* =  − 0.174**) and between duration and Q6 only (*r* =  − 0.183**, *P* < 0.001).

These results are in line with Mattsson et al.’s [9] results which found that the SEMCD-Swe was moderately correlated with pain (HAQ VAS, RAND-36), skin tightness (mRSS), and severity of organ affection of peripheral vascular system, lung, and kidney (MSS) of SSc disease; moreover, the SEMCD-Swe was found to have a weak correlations with disease duration. Also these results matched with Gruber-Baldini et al. [22] as regards weak correlation between the SEMCD and duration of the disease in other chronic conditions. However, as regards disease duration, our results mismatched with Allama et al.’s which revealed significant positive correlation with duration of diabetes mellitus (DM) [13] that can be explained by short disease duration of in the patient of our study (median = 6).

Test–retest reliability and internal consistency were estimated in patients (ICC ranged from 0.87 to 0.997) indicating excellent agreement and high reproducibility between the SEMCD questions. Also, internal consistency is acceptable as the value of Cronbach’s alpha ranged from 0.660 to 0.78. Also, the correlations of item-total ranged from 0.811 to 0.917 indicative to a good reliability. These findings are in line with Mattsson et al.’s [9] results (Cronbach’s alpha 0.85), and the item-total correlation ranged from 0.50 to 0.69.

## Limitations

Our study faced limitations where the selection bias of patients was found as not all patients attending our medical institutes have good reading skills. Also SEMCD questionnaire was not evaluated in RA patients in other country and this added more limitation to our study. Moreover, this questionnaire results are from the patient perspective and reflect outcomes that are important for the patient rather than those that are represent a priority for a healthcare professionals.

## Conclusion

This study has confirmed that the Arabic version of the SEMCD questionnaire can be used as a valid and reliable tool as a measure for assessment of patient’s self-efficacy in management of RA as a chronic disease regarding construct validity, internal consistency, and reliability, that it is, to our knowledge, the first time to be evaluated in RA.
